# Computed Tomography of *Coenurus cerebralis* in Sheep: High Diagnostic Sensitivity With Limited Association to Clinical Severity

**DOI:** 10.1155/vmi/3070740

**Published:** 2026-08-31

**Authors:** Javad Abbasi, Amin Zamani, Majid Masoudi Fard, Mostafa Abdollahi, Arezoo Ramezani, Amin Anoushepour, Danial Goli

**Affiliations:** ^1^ Department of Animal and Poultry Health and Nutrition, Faculty of Veterinary Medicine, University of Tehran, Tehran, Iran, ut.ac.ir; ^2^ Institute of Biomedical Research, University of Tehran, Tehran, Iran, ut.ac.ir; ^3^ Department of Veterinary Clinical Sciences, Ka.C., Islamic Azad University, Karaj, Iran, azad.ac.ir; ^4^ Department of Radiology and Surgery, Faculty of Veterinary Medicine, University of Tehran, Tehran, Iran, ut.ac.ir; ^5^ Department of Clinical Sciences, Faculty of Veterinary Medicine, Semnan University, Semnan, Iran, semnan.ac.ir

**Keywords:** brain imaging, clinical severity, Hounsfield unit, intracranial cysts, lesion localization, molecular diagnosis

## Abstract

**Background and Aims:**

Coenurosis is an important cause of neurological disease in sheep in endemic regions. Ante‐mortem diagnosis is primarily based on clinical signs, which are often nonspecific. Computed tomography (CT) can detect intracranial cystic lesions. This study aimed to describe CT imaging features of *Coenurus cerebralis* in sheep and to evaluate their association with neurological clinical findings.

**Methods:**

Twenty 7‐month‐old Afshari male lambs with neurological signs consistent with coenurosis and 20 healthy matched controls were included. Affected animals underwent neurological examination, clinical severity scoring, and cranial CT imaging using a standardized protocol. Cyst number, volume, and anatomical location were recorded, and Hounsfield unit (HU) values of cyst fluid, brain parenchyma, and skull bone were measured. Diagnosis was confirmed by gross pathological examination and PCR targeting the mitochondrial COX1 gene. Associations between CT variables and clinical findings were analyzed using regression models.

**Results:**

All cysts identified at necropsy were detected by CT, corresponding to a sensitivity of 100%. No significant associations were found between cyst number, size, or location and neurological signs or clinical severity score (*p* > 0.05). HU values of brain parenchyma and cranial bone were significantly lower in affected sheep compared with controls (*p* < 0.001).

**Conclusion:**

CT is highly sensitive for detecting cerebral cysts in sheep with coenurosis. However, CT‐derived structural features are not associated with clinical severity and should not be used for prognostic evaluation. CT remains a valuable adjunct diagnostic tool when interpreted alongside clinical findings.

## 1. Introduction

Coenurosis is a parasitic disease caused by the larval stage (*Coenurus cerebralis* or *C. cerebralis*) of the cestode *Taenia multiceps* and primarily affects small ruminants, particularly sheep and goats. The parasite’s life cycle involves canids, mainly domestic dogs, as definitive hosts that shed eggs in their feces, contaminating pastures and water sources. Following ingestion of infective eggs, sheep act as intermediate hosts in which oncospheres penetrate the intestinal wall, enter the bloodstream, and migrate to the central nervous system (CNS), where they develop into cystic lesions known as *C*. *cerebralis*. These cysts gradually enlarge over weeks to months, causing compression of neural tissues and progressive neurological dysfunction [1].

Coenurosis is endemic in many regions of the world, including Africa, the Middle East, Southern Europe, and Asia [2–4]. The disease predominantly affects young lambs, particularly those less than 12 months of age, owing to early exposure to contaminated grazing areas and their relatively immature immune system [2].

Clinically, coenurosis occurs in acute and chronic forms. The acute form is associated with the migration of large numbers of oncospheres through the brain parenchyma and may result in depression, anorexia, weight loss, blindness, and progressive weakness. In contrast, the chronic form develops following the establishment of space‐occupying coenurus cysts within the brain. Clinical manifestations include head tilt, blindness, ataxia, circling, recumbency, seizures, and torticollis and are traditionally considered to be influenced by the number, size, and anatomical location of the cysts [3, 4].

Coenurosis is associated with substantial economic losses in small‐ruminant production systems due to reduced productivity, premature culling, treatment expenses, and animal welfare concerns [5]. The chronic nature of the disease and the challenges associated with ante‐mortem diagnosis further increase its economic impact, particularly in endemic regions [5, 6].

Current diagnostic approaches, including clinical examination and serological testing, have limited sensitivity and specificity. Although magnetic resonance imaging (MRI) provides excellent soft‐tissue contrast, its routine application in veterinary practice is often restricted by cost and limited availability [6]. Computed tomography (CT) offers a practical alternative because of its rapid image acquisition, wider accessibility, and ability to objectively assess cyst size, location, and tissue attenuation characteristics [7]. However, the relationship between CT‐derived imaging parameters and clinical manifestations in sheep remains poorly understood.

Previous studies have described CT findings in ovine coenurosis; however, most have focused primarily on lesion detection and descriptive imaging characteristics rather than evaluating quantitative imaging variables in relation to standardized neurological severity scores. In addition, molecular confirmation has not consistently been incorporated into studies investigating imaging‐clinical associations. Therefore, the present study aimed to quantitatively evaluate CT‐derived cyst characteristics and investigate their association with standardized clinical severity scores in sheep with molecularly confirmed coenurosis.

## 2. Materials and Methods

### 2.1. Study Area

**FIGURE 1:**
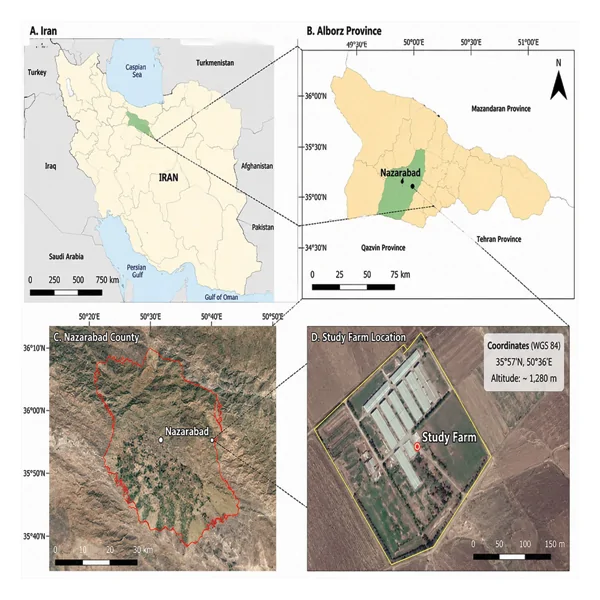
Location of the study area. (A) Iran with Alborz province highlighted; (B) Alborz province with Nazarabad county; (C) Nazarabad county; and (D) location of the study farm.

The study was conducted on a semi‐intensive sheep farm located in Nazarabad County, Alborz Province, Iran (35°57′N, 50°36′E) (Figure 1). The region is characterized by a semiarid climate, with hot summers, cold winters, and an average annual rainfall of approximately 320 mm.

### 2.2. Study Animals

The study population was selected from a semi‐intensive flock consisting of approximately 6000 Afshari sheep with a documented history of coenurosis. All animals were maintained under similar husbandry and feeding conditions throughout the study period.

The sample size was determined based on previous studies [7, 8]. A total of 40 male Afshari lambs, all 7 months of age, were enrolled in the study. Animals were initially screened based on flock history and neurological examination findings. Twenty lambs exhibiting progressive neurological signs consistent with cerebral coenurosis were assigned to the case group. Clinical suspicion was subsequently supported by CT and confirmed by gross pathological examination and polymerase chain reaction (PCR) identification of *C. cerebralis*. Twenty clinically healthy lambs from the same flock, matched for age, sex, breed, and management conditions, were selected as the control group. Control animals exhibited no neurological abnormalities on clinical examination and had no history of neurological disease.

All enrolled animals underwent a standardized clinical examination before inclusion in the study. Animal identification number, age, sex, clinical history, and neurological findings were recorded for each animal. Lambs were included in the case group if they were 7 months of age, exhibited progressive neurological signs for more than 7 days, had clinical suspicion of cerebral coenurosis based on neurological examination and flock history, and had cerebral cysts confirmed by CT, gross pathological examination, and PCR. Animals were excluded if there was clinical or historical evidence of listeriosis, polioencephalomalacia (PEM), cranial trauma, intracranial abscessation, otitis media or interna, or administration of corticosteroids within 14 days before clinical evaluation.

### 2.3. Clinical Assessment

All animals enrolled in the study underwent a thorough clinical examination, and their history, identification number, sex, and age were recorded. Clinical status was scored according to the criteria outlined in Table 1. This scoring system was adapted from previous clinical assessment methods and modified to reflect the spectrum of neurological signs observed in ovine coenurosis.

**TABLE 1:** Neurologic severity scoring system used for clinical status scoring.

Score	Description	Normal mention	Normal gait	Menace reflex	Ataxia	Circling	Recumbency	Head deviation	Seizure
0	Normal	+	+	+	−	−	−	+/−	−
1	Mild	+	−	+	+/−	−	−	+/−	−
2	Moderate	−	−	+/−	+	+	−	+/−	−
3	Severe	−	−	−	+	−	+	+/−	+/−

### 2.4. CT Examination

An intravenous catheter was placed in the saphenous vein of each sheep to facilitate the administration of anesthetic agents and fluids. For premedication, atropine (0.2 mg/kg) and diazepam (0.2 mg/kg) were administered intravenously, followed by induction of anesthesia with ketamine (3 mg/kg IV). Animals were positioned in sternal recumbency, and dorsoventral CT images of the head were acquired. No anesthesia‐related complications were observed.

CT examinations were performed using a multislice CT scanner (Somatom Spirit 2; Siemens, Munich, Germany). Scanning parameters included a tube rotation time of 1.5 s, a slice thickness of 2 × 1 mm, a tube current of 75–95 mAs, a tube voltage of 130 kVp, and a pitch of 1.95. Scanning extended from the nasal tip to the occipital bone to ensure complete evaluation of intracranial structures. Brain window settings (window width, 80; window level, 40) and skull window settings (window width, 2000; window level, 400) were applied for optimal visualization and characterization of intracranial structures.

**FIGURE 2:**
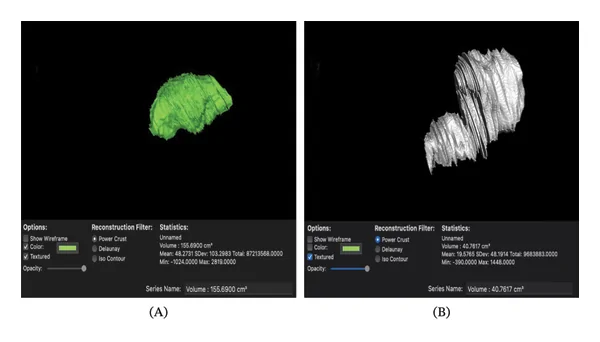
Volume measurements using Horos software: (A) total brain volume and (B) cyst volume.

**FIGURE 3:**
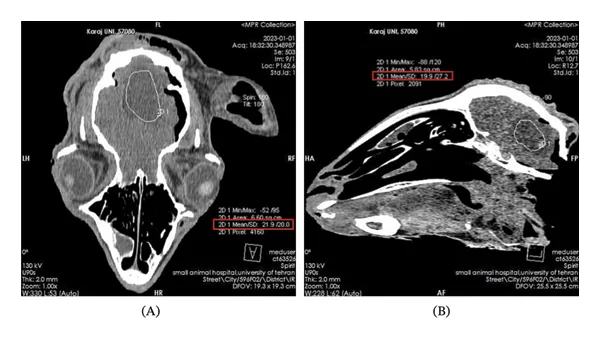
Measurement of the HU of the cyst in a patient using Leonardo software. (A) Axial plane. (B) Sagittal plane.

Volumetric analyses and HU measurements were performed using the Leonardo workstation and Horos software (Version 3.3.4) (Figures 2 and 3).

All CT images were independently evaluated by three observers, and ROI placement was standardized across all animals. High interobserver agreement was observed for cyst identification and volume measurements, with an intraclass correlation coefficient (ICC) of 0.92, indicating excellent agreement.

### 2.5. Gross Pathological Examination

**FIGURE 4:**
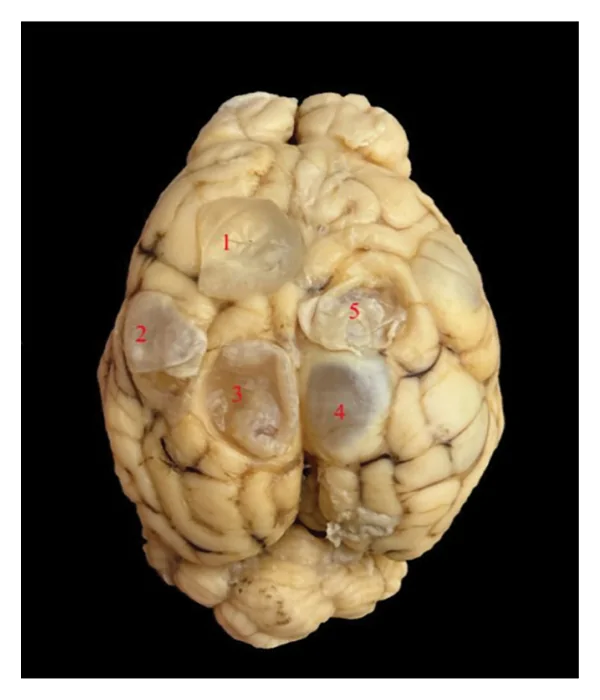
Five *C*. *cerebralis* cysts in the cortical region.

**FIGURE 5:**
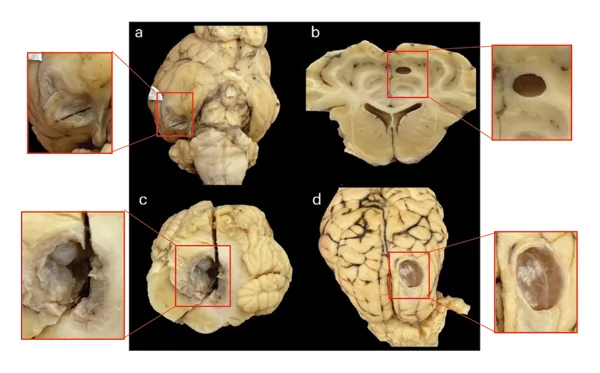
*C*. *cerebralis* cysts.

**FIGURE 6:**
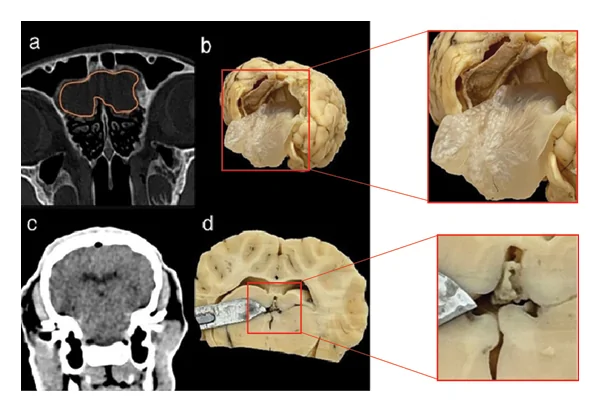
Review of the consistency of CT findings in gross pathology: (a, b) A cyst in the frontal lobe; (c, d) A cyst in the third ventricle and parietal lobe.

**FIGURE 7:**
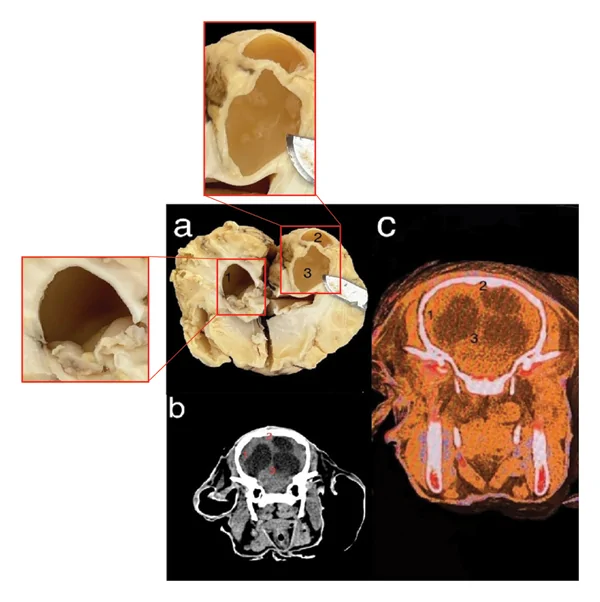
Review of the consistency of CT findings in gross pathology: (a) necropsy view of *C*. *cerebralis* cysts; (b) two‐dimensional CT slice; and (c) 3D reconstructed CT image.

Case‐group animals were culled for commercial purposes. Selected heads were fixed in 10% buffered formalin. Gross necropsy was performed to correlate CT findings with macroscopic lesions (Figures 4 and 5). Cyst localization and characteristics were systematically recorded and compared between imaging and necropsy observations (Figures 6 and 7). Gross pathological examination was considered the reference standard for confirmation of intracranial cyst presence and localization, as direct visualization of cystic lesions remains the definitive postmortem diagnostic method for ovine coenurosis.

### 2.6. PCR and Sequence Analysis

All brain cysts identified in animals of the case group were aseptically aspirated for molecular confirmation. Genomic DNA was extracted using a commercial tissue DNA extraction kit (SinaClone, Iran) according to the manufacturer’s instructions. DNA concentration and purity were assessed spectrophotometrically (Bio‐Rad, USA), and extracted DNA samples were stored at −20°C until analysis.

PCR amplification targeted the mitochondrial cytochrome c oxidase subunit 1 (COX1) gene of *Taenia multiceps* using the primer pair T.F (5′‐TTTTTTGGGCATCCTGAGGTTTAT‐3′) and T.R (5′‐TAAAGAAAGAACATAATGAAAATG‐3′), previously validated for the molecular detection of *T. multiceps* [9]. Each 50 μL reaction mixture contained 10 mM Tris‐HCl, 50 mM KCl, 1.5 mM MgCl_2_, 0.2 mM dNTPs, 20 pmol of each primer, and 2 U of Taq DNA polymerase (SinaClone, Iran). Amplification was performed with an initial denaturation at 94°C for 5 min, followed by 35 cycles of denaturation at 94°C for 30 s, annealing at 55°C for 30 s, and extension at 72°C for 30 s, with a final extension at 72°C for 5 min. Negative controls were included in all PCR runs. Amplified products were separated by electrophoresis on 1% agarose gels and visualized by ethidium bromide staining under ultraviolet light [9].

PCR amplification confirmed that all cerebral cysts collected from the 20 animals in the case group were caused by *Taenia multiceps* (*C*. *cerebralis* larval stage).

Positive PCR products were purified and subjected to bidirectional sequencing (Codon Genetic Group, Tehran, Iran). The resulting sequences were deposited in GenBank under accession number PP472080.1. Sequence identity was verified by BLAST analysis against reference sequences available in the GenBank database. All isolates showed the highest sequence similarity to *Taenia multiceps* and exhibited no detectable sequence variation among isolates. Consequently, no analysis of associations between parasite genotype and clinical findings could be performed.

### 2.7. Ethical Approval

All procedures involving animals were conducted in accordance with institutional guidelines for the care and use of animals in research. Ethical approval for the study was obtained from the Ethics Committee of the Faculty of Veterinary Medicine, University of Tehran (Approval No. 213/43/1403).

Animal handling, anesthesia, and CT imaging procedures were performed in compliance with applicable animal welfare regulations to minimize stress and discomfort. Animals included in the case group were culled as part of routine commercial slaughter practices and not specifically for the purposes of this study.

### 2.8. Statistical Analysis

Statistical analyses were performed using SPSS software (Version 22.0; IBM Corp., Armonk, NY, USA). CT measurements and clinical assessments were recorded independently by different investigators and matched during statistical analysis using individual animal identification codes to minimize observer bias. Data distribution was assessed using the Shapiro–Wilk test. Because HU values were not normally distributed, comparisons between the case and control groups were performed using the Mann–Whitney *U* test.

The primary explanatory variables included cyst count, cyst size, and affected brain lobe. These variables were selected a priori based on their biological relevance and the objectives of the study rather than by statistical screening procedures. Univariable regression analyses were initially performed to evaluate the independent association of each CT‐derived variable with the clinical outcomes. Subsequently, all predefined explanatory variables were entered simultaneously into the multivariable regression model using the Enter method; therefore, no *p* value‐based variable selection criterion was applied.

Binary logistic regression was used to evaluate associations between CT‐derived variables and dichotomous neurological outcomes, whereas ordinal logistic regression was used to assess associations with the clinical severity score. Because only three biologically relevant explanatory variables were included and all were predefined, formal confounder selection procedures were not performed. Similarly, assessment of multicollinearity was not considered necessary because of the limited number of predefined predictors and the absence of conceptual overlap among them. Statistical significance was defined as *p* < 0.05. Where estimable, odds ratios (ORs) with 95% confidence intervals (CIs) were reported.

## 3. Results

### 3.1. Diagnostic Sensitivity of CT

The diagnostic sensitivity of CT for detecting intracranial coenurus cysts in sheep was evaluated using gross pathological examination as the reference standard. All cysts identified during gross pathological examination were detected on CT images, resulting in an observed sensitivity of 100% (95% CI: 92.1%–100%). Interobserver agreement demonstrated excellent reliability of CT measurements, with an ICC of 0.92 for cyst volume assessment.

### 3.2. Clinical Findings and CT Correlation

All enrolled lambs were 7 months of age; therefore, age‐related comparisons were not applicable.

Binary logistic regression analyses were performed to assess whether cyst count, cyst size, or affected brain lobe could predict individual neurological signs in sheep with *C*. *cerebralis* infection. No statistically significant associations were identified between CT‐derived variables and neurological outcomes, including recumbency (*p* = 0.30), circling (*p* = 0.20), seizures (*p* = 0.20), head deviation (*p* = 0.80), blindness (*p* = 0.50), abnormal mentation (*p* = 0.60), and abnormal gait (*p* = 0.60).

Due to sparse outcome distributions and model instability resulting from quasi‐complete separation, ORs and corresponding 95% CIs could not be estimated reliably and were therefore not reported.

### 3.3. Clinical Scoring and CT

As shown in Table 2, univariable ordinal logistic regression analyses revealed no significant associations between clinical severity score and either cyst count (*p* = 0.70) or affected brain lobe (*p* = 0.90). For cyst size, the model did not produce reliable estimates because of quasi‐complete separation (NE in Table 2). Similarly, in the multivariable ordinal regression model that included all three predictors, parameter estimates were not reliably estimable for the same reason (NE in Table 2). Collectively, these findings indicate that CT‐derived measures of cyst burden, whether evaluated individually or in combination, do not reliably predict the overall clinical severity of ovine coenurosis.

**TABLE 2:** Association between CT variables and clinical severity score.

Variable/model	OR (95% CI)	*p* value
Univariable model		
Cyst count	1.87 (0.05–74.6)	0.70
Cyst size	NE	NE
Affected lobe	1.82 (0.21–16.15)	0.90
Multivariable model	NE	NE

*Note:* NE, not estimable due to quasi‐complete separation.

### 3.4. HU Analysis

As HU measurements were not normally distributed according to the Shapiro–Wilk test, comparisons between groups were performed using the Mann–Whitney *U* test. Significant differences were observed in both brain parenchymal and skull bone HU values between affected and control sheep (*p* < 0.001 for both variables) (Table 3).

**TABLE 3:** HU in studied groups (mean ± SD).

Tissue	Control group				Case group			
	Mean	Max	Min	Median	Mean	Max	Min	Median
Cyst fluids	—	—	—	—	16.95 ± 2.11	23.28	10.62	16.67
Brain parenchyma	50.20 ± 2.71^a^	53.12	48.94	49.70	45.01 ± 3.48^a^	49.89	38.65	45.00
Skull bone	588.30 ± 16.10^a^	637	540	572.30	448.40 ± 9.33^a^	476.40	421.32	442.78

^a^Dissimilar letters indicate significant differences (*p* < 0.05).

Affected sheep exhibited significantly lower brain parenchymal and skull bone HU values than healthy controls. HU values for cyst fluid were recorded only in the case group because no cystic structures were present in the control animals.

Additionally, cerebrospinal fluid (CSF) HU values were not evaluated because the ventricular system in sheep is anatomically narrow and poorly delineated on noncontrast CT images. Combined with the limited soft‐tissue contrast resolution of CT, this prevented reliable identification of ventricular CSF spaces and accurate HU measurement under the imaging protocol used in the present study.

## 4. Discussion

CT is a valuable adjunct imaging modality for the evaluation of intracranial lesions in sheep with suspected coenurosis. It enables rapid, noninvasive visualization of cystic structures within the brain and supports clinical diagnosis in cases where neurological examination alone is insufficient for lesion localization [10]. In the present study, CT identified all intracranial cysts that were subsequently confirmed by gross pathological examination, yielding an observed sensitivity of 100% within the study population. This finding is consistent with previous reports describing CT as a reliable method for detecting intracranial cystic lesions in ovine coenurosis [7, 8, 10–13]. However, CT alone cannot reliably differentiate *C*. *cerebralis* from other intracranial cystic conditions, such as cerebral hydatidosis, and therefore its interpretation should always be integrated with confirmatory diagnostic methods, including pathological or molecular evaluation [11].

Despite accurate lesion detection, no significant association was observed between CT‐derived variables (including cyst number, size, and anatomical location) and neurological severity or individual clinical signs. This suggests that gross structural lesion characteristics visible on CT do not adequately explain the variability in clinical presentation among affected animals. Neurological manifestations are likely influenced by a combination of lesion location, local tissue response, intracranial pressure dynamics, and individual host variability, which cannot be fully characterized using structural CT imaging alone [14–18].

Significant differences in Hounsfield unit (HU) values of brain parenchyma and cranial bone were observed between affected and healthy sheep. These findings indicate measurable imaging differences between groups and may reflect alterations associated with the presence of intracranial cysts. However, because CT attenuation values do not directly represent underlying pathological mechanisms, these observations should be interpreted strictly as descriptive imaging findings [19–21].

From a clinical perspective, CT should be considered primarily a diagnostic and lesion‐localization tool rather than a prognostic indicator. It provides valuable anatomical information that may assist in decision‐making in referral settings and may contribute to future surgical planning when intervention is considered. Nevertheless, its use should remain supportive and always be interpreted in conjunction with neurological examination findings.

This study has several limitations. The relatively small sample size limited statistical power and may have contributed to instability in statistical modeling. The study population was restricted to a single flock of Afshari sheep from one geographic region, which may limit generalizability. In addition, CT findings were not compared with MRI, which prevented evaluation of relative diagnostic performance between imaging modalities. Furthermore, CT alone cannot definitively distinguish coenurosis from other cystic intracranial diseases without molecular or pathological confirmation. The absence of histopathological assessment also limits detailed tissue‐level correlation. Finally, the study was limited to structural imaging and clinical scoring, without functional or longitudinal follow‐up assessment.

## 5. Conclusion

CT is a highly sensitive imaging modality for the detection and localization of *C. cerebralis* cysts in sheep. However, CT‐derived structural characteristics were not associated with neurological severity and should not be considered prognostic indicators. Clinical evaluation remains essential, and CT should be interpreted as a complementary diagnostic tool for lesion localization and confirmation. In referral centers, CT may facilitate clinical decision‐making and preoperative assessment, while in research settings it provides an objective method for characterizing intracranial lesions.

## Author Contributions

Amin Zamani contributed to the conception of the study, sample collection, CT imaging, data interpretation, and manuscript drafting and revision. Javad Abbasi supervised the study, contributed to project design, oversight, and final approval of the manuscript. Majid Masoudi Fard contributed to supervision and validation of CT imaging and critically revised the manuscript. Mostafa Abdollahi contributed to manuscript editing and scientific input. Arezoo Ramezani participated in CT imaging procedures and manuscript revision. Amin Anoushepour contributed to clinical case identification, supervision of the study, and approval of the final manuscript. Danial Goli contributed to data collection and field coordination.

## Funding

No funding was received for this manuscript.

## Disclosure

All final content and interpretations are solely the responsibility of the authors.

## Conflicts of Interest

The authors declare no conflicts of interest.

## Data Availability

The data that support the findings of this study are available from the corresponding author upon reasonable request.
